# Rotating Surfaces Promote the Shedding of Droplets

**DOI:** 10.34133/research.0023

**Published:** 2023-01-10

**Authors:** Ran Tao, Wei Fang, Jun Wu, Binhong Dou, Wanghuai Xu, Zhanying Zheng, Bing Li, Zuankai Wang, Xiqiao Feng, Chonglei Hao

**Affiliations:** ^1^ School of Mechanical Engineering and Automation, Harbin Institute of Technology, Shenzhen 518055, China.; ^2^ Institute of Biomechanics and Medical Engineering, Applied Mechanics Laboratory, Department of Engineering Mechanics, Tsinghua University, Beijing 100084, China.; ^3^ Department of Mechanical Engineering, City University of Hong Kong, Hong Kong 999077, China.

## Abstract

Achieving rapid shedding of droplets from solid surfaces has received substantial attention because of its diverse applications. Previous studies have focused on minimizing contact times of liquid droplets interacting with stationary surfaces, yet little consideration has been given to that of moving surfaces. Here, we report a different scenario: A water droplet rapidly detaches from micro/nanotextured rotating surfaces in an intriguing doughnut shape, contributing to about 40% contact time reduction compared with that on stationary surfaces. The doughnut-shaped bouncing droplet fragments into satellites and spontaneously scatters, thus avoiding further collision with the substrate. In particular, the contact time is highly dependent on impact velocities of droplets, beyond previous descriptions of classical inertial-capillary scaling law. Our results not only deepen the fundamental understanding of droplet dynamics on moving surfaces but also suggest a synergistic mechanism to actively regulate the contact time by coupling the kinematics of droplet impingement and surface rotation.

## Introduction

Analogous to a free oscillation system, water droplets bounce off spontaneously when impacting on superhydrophobic surfaces [1,2]. Indeed, the phenomenon of liquid shedding is omnipresent in nature (e.g., lotus leaves or water striders) [3,4] and has potential implications in a wide spectrum of industrial applications, such as anti-icing/fogging [5–10], electricity generation [11,12], fluid transport [13], heat transfer [14–16], drag reduction [17], and self-cleaning [18–21]. To characterize the quality of liquid shedding, how long the liquid droplet is in contact with the solid becomes an important issue. In this context, the concept of contact time *τ* has emerged, which is defined as the interval between moments that a droplet first lands on the substrate and that it completely detaches from the surface. Previous studies reported that *τ* scales with the inertial-capillary time scale $\tau^{'}={\left(\rho D_{0}^{3}/\gamma \right)}^{1/2}$, which is independent of the impact velocity [22]. Here, *ρ*, *D*_0_, and *γ* represent the mass density, diameter, and surface tension of the liquid droplet, respectively.

The contact time is associated with the efficiency of mass, momentum, and energy transfer between the liquid and the underlying solid. Thus, it is usually beneficial to minimize *τ* in many practical applications [5,23]. In the past decade, various strategies have been exploited to go beyond the limit of *τ* via well-designed structures to remodel the solid–liquid interactions [24–31]. These studies put emphasis on reducing *τ* on stationary substrates exclusively, whereas a broad range of systems in both nature and industrial engineering necessitate surface motion to achieve diverse functionalities. Thus, a comprehensive understanding of droplet dynamics on moving substrates is highly expected [32–34]. In our daily scene, translation and rotation are 2 ubiquitous forms of motion [35]. Recent studies have revealed that translational moving surfaces enable the reduction of *τ* by imparting droplet with asymmetric bouncing [36,37]. However, the scenario involving rotating surfaces, an equally important counterpart, is still poorly understood.

Here, we combine experiments, theoretical analysis, and numerical simulations to study surface rotating effect on droplet dynamics, which is completely beyond previous research focusing on surface translational motion effect [33,37–39]. We observed that the droplet bounces in a doughnut-shaped configuration, which renders a rapid liquid shedding from rotating surfaces. With the introduction of new kinetic parameters (i.e., angular velocity), the conventional inertial-capillary time scale is no longer applicable. Instead, we reveal that *τ* is regulated by both the droplet impact velocity *v*_i_ and the substrate angular velocity *ω*. The generality of the contact time reduction on rotating superhydrophobic surfaces is also examined by conducting experiments with different droplet sizes, substrate materials/structures, and landing locations.

## Results and Discussion

### Droplet bouncing dynamics

**Fig. 1.**
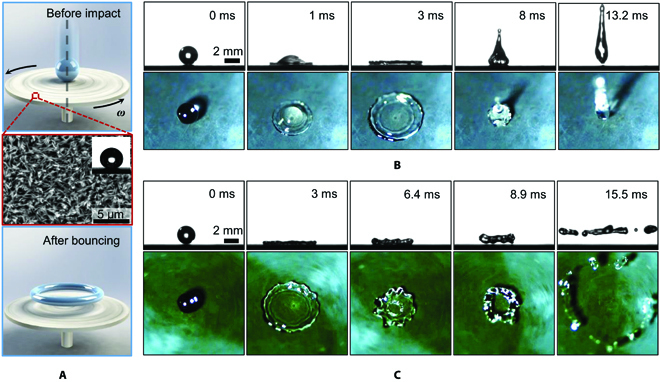
Comparison of droplet impact dynamics on stationary and high-speed rotating surfaces. (A) The schematic of key elements of experimental setup for doughnut-shaped bouncing. A water droplet impacts on the center of a high-speed rotating sample with angular velocity *ω* (top). The surfaces of the sample are decorated by nanostructured copper oxide exhibiting high superhydrophobicity (middle). The droplet bounces off the surfaces in a doughnut shape (bottom). (B and C) Selected snapshots captured by high-speed cameras from both top view and side view showing the droplet impact dynamics with a velocity *v*_i_ = 1.56 m/s on (B) a stationary surface where the droplet exhibits a typically conventional rebound with contact time *τ* ~ 13.2 ms and (C) a rotating surface (*ω* = 9,000 rpm) where the droplet bounces in a doughnut shape, along with a contact time *τ* of 8.9 ms.

In the experiments, we release water droplets from a predetermined height to impact on the center of a superhydrophobic surface rotating with an angular velocity *ω* (Fig. 1A). The substrate is a thin copper disc covered with copper oxide (CuO), which owns knife-like nanostructures (Fig. 1A, inset). The whole sample is further treated with fluorosilane to render its superhydrophobicity with an apparent contact angle of 161° ± 1° (see Methods and Fig. S1). The advancing and receding contact angles are measured to be 164° ± 3° and 157° ± 1°, respectively.

Figure 1B and C illustrates the striking contrast of droplet impact dynamics (with size *D*_0_ ≈ 2.5 mm, velocity *v*_i_ ≈ 1.57 m/s) on stationary (*ω* = 0 rpm) and rotating (*ω* = 9,000 rpm) superhydrophobic surfaces, filmed from both the side view and top view using 2 synchronized high-speed cameras. As expected, on the stationary control surface, a regular impingement behavior involving symmetrical spreading, retraction, and bouncing off is observed (Fig. 1B), and the total solid–liquid contact time is measured to be about 13.2 ms, in agreement with previous works [27,40]. Distinct from typical dewetting behavior observed on stationary surfaces, the droplet impacting on a high-speed rotating surface gets punctured from the center during the retraction process and forms a hole, which brings in an additional inner rim that recedes with the outer rim simultaneously (Fig. 1C and see Movies S1 and S2). When the 2 rims collide with each other, it yields a vertical momentum that is sufficient to lift off the droplet in a symmetric doughnut-shaped configuration (also schematically depicted in the bottom panel of Fig. 1A). The synchronized side-view images clearly suggest a distinct *τ* of about 8.9 ms, engendering a pronounced contact time reduction compared with the stationary surface, as an additional contact line is involved in the retraction stage. Our observation manifests a remarkable contact time reduction without breaking the radial symmetry, showing a striking contrast with previous studies on translational moving surfaces [36,37], which was caused by the asymmetric droplet receding behavior. Although the droplet is subjected to zero resultant force, the net momentum is nonzero. Therefore, the doughnut-shaped droplet continues to rotate and expand in air after bouncing off the surface, finally disintegrating into a couple of satellite droplets due to Plateau-Rayleigh instability. These satellite droplets scatter radially, thus avoiding further contact with underlying substrate. Together, the rotating surface is capable of substantially reducing the liquid–solid interaction and taking away the bouncing droplet spontaneously, thus exhibiting an excellent liquid repellency performance. We also perform experiments using hydrophilic rotating surfaces. As shown in Fig. S2, the drop spreads into a thin liquid film along the surface after impact and then disintegrates into satellite drops near the triple-phase contact line, without further receding and bouncing behaviors due to their high surface energy properties.

### Experimental characterization

**Fig. 2.**
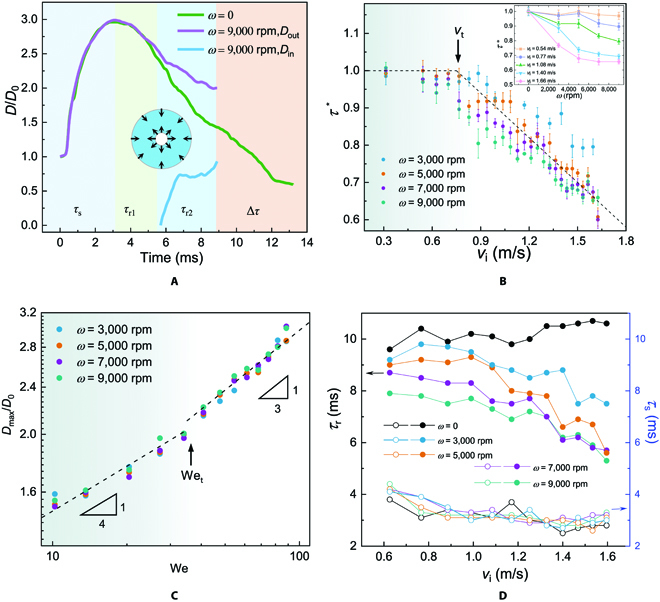
Characterization of droplet impact on rotating surfaces. (A) Comparison between the temporal evolutions of droplet spreading length on a stationary (*ω* = 0 rpm) and a rotating (*ω* = 9,000 rpm) substrate. The inset illustrates the schematic of the droplet retraction path, where the inner and outer liquid rims recoil simultaneously. (B) Normalized contact time τ^*^ as a function of droplet impact velocity *v*_i_ for various angular velocities *ω* between 3,000 and 9,000 rpm. The inset replots the data to show the variation of τ^*^ with *ω*. (C) The normalized maximum spreading diameter as a function of the Weber number, with scaling behavior of We^1/4^ and We^1/3^ depicted in the left and right regimes, respectively. (D) Variations of the spreading time *τ*_s_ (open symbols) and retraction time *τ*_r_ (solid symbols) with respect to the impact velocity *v*_i_, demonstrating that the contact time reduction behavior on rotating surfaces is dominated by the solid–liquid interplay during droplet retracting stage.

To gain insights into the physical mechanisms underlying the observed contact time reduction, we further compare the temporal evolution of droplet spreading length *D*, normalized by its initial diameter *D*_0_ for the 2 cases discussed above, as shown in Fig. 2A. Here, we use the top-view projected rim to quantify the spreading length, which is slightly different from the actual contact line profile. Careful inspection of droplet dynamics on the stationary surface (green line in Fig. 2A) shows that the droplet first spreads with an average velocity of ~2.47 m/s to a maximum of ~3 times its initial diameter and then retracts with an estimated velocity of 0.58 m/s until fully bouncing off from the surface at around 13.2 ms.

Unlike the regular bouncing on stationary surfaces, the droplet dynamics on rotating surfaces is more intricate. Specifically, the variation of the dimensionless spreading length *D*/*D*_0_ can be divided into 3 regimes. Over the first period of *τ*_s_, the droplet spreads to its maximum length with an approximate velocity of 2.49 m/s (*D*_out_; violent line in Fig. 2A), identical to that on the stationary surface. It can be understood that the spreading dynamics of an impacting droplet is dominated by inertia [41], during which the rotation effect of the underlying substrate is negligible. In the second regime over the period of *τ*_r1_, the droplet starts to retract at an averaged velocity of 0.44 m/s, slightly slower than that measured on the stationary counterpart due to the outward centrifugal force imparted by the rotating surface. Then, at the beginning of the third period *τ*_r2_, a singularity in the retracting liquid film appears from the center, provokes a hole nucleation, and leads to a newly generated inner contact line retracting toward the outer rim simultaneously (*D*_in_; blue line in Fig. 2A). The inset of Fig. 2A illustrates a schematic of synergistic recoiling path of the inner and outer rims. The initial retracting velocity of the inner rim is measured to be ~2.14 m/s, which is much higher than that of the outer rim. This unbalanced feature in terms of retraction velocities can be attributed to the nonuniform thickness profile between the lamella center and the edge of the spreading droplet. Thus, the overall contact time of droplet bouncing on rotating surfaces is calculated as *τ = τ*_s_ + *τ*_r1_ + *τ*_r2_, which causes its reduction by Δ*τ* compared with that on stationary surfaces. Moreover, the lifting-off droplet continues to expand as evidenced by increasing outer rim diameter *D*_out_ with time (Fig. S3), suggesting a centrifugal force dominated kinematics and a spontaneous liquid removal behavior.

To better understand the reduction of contact time, we systematically investigate the effect of kinetic parameters over a wide range of *v*_i_ from 0.31 to 1.71 m/s and *ω* from 0 to 9,000 rpm, respectively. Basically, the behavior of contact time reduction occurs within appropriate combinations of *v*_i_ (or Weber number) and *ω*, beyond which either conventional bouncing or splashing appears (Fig. S4). Here, the Weber number (We) is defined as $\text{We}=\rho v_{i}^{2}D_{0}/\gamma$. The characterized *τ* in the contact time reduction region is shown in Fig. S5. By replotting the data in Fig. 2B, we present the variation of normalized contact time τ^*^ = *τ* / *τ*^0^ as a function of impact velocity *v*_i_, where *τ*^0^ denotes the corresponding contact time on the stationary surfaces. It clearly shows that the data collapse into 2 different regimes, which are separated by a threshold velocity *v*_t_. Note that *v*_t_ slightly fluctuates over a narrow range from about 0.7 to 0.94 m/s (corresponding to threshold We_t_ of 17 to 31) that depends on the angular velocity *ω*. Briefly, in the regime of lower velocity (*v*_i_ < *v*_t_), τ^*^ plateaus to unity, indicating that the rotating effect on the reduction of contact time is negligible. This observation can be understood that the slight deformation of impacting droplet at low *v*_i_ results in a limited solid–liquid contact area and, thus, the interplay between the droplets and underlying substrate is trifling. On the other hand, the trend is no longer valid in the regime of higher velocity (*v*_i_ > *v*_t_); instead, τ^*^ is monotonically decreased with increasing *v*_i_, resulting in the contact time reduction up to ~40%. Therefore, the conventional inertial-capillary scaling of ${\left(\rho D_{0}^{3}/\gamma \right)}^{1/2}$ is not applicable to the observed unique phenomenon anymore, due to the introduction of a new physical parameter *ω* that essentially alters the liquid–solid interaction and reshapes the liquid hydrodynamics upon drop impact. Replotting τ^*^ as a function of *ω* in the inset of Fig. 2B explicitly signifies the importance of the rotating surface in governing the contact time. In addition, we also show the normalized maximal spreading diameter *D*_max_/*D*_0_ of the impacting droplet as a function of We, as shown in Fig. 2C. We observe the scaling behavior of *D*_max_/*D*_0_ ~ We^1/4^ in the regime of We < We_t_, which has been reported for droplets impacting on stationary surfaces [41]. However, the scaling does not fit the data well in the regime of We > We_t_. Alternatively, we find our results show good agreement with *D*_max_/*D*_0_ ~ We^1/3^, which might be intervened by the rotation effect from the underlying substrate.

By decomposing the droplet dynamics into separate spreading and retraction stages, we further plot the spreading time *τ*_s_ and retraction time *τ*_r_ as functions of impact velocity *v*_i_, respectively. As shown in Fig. 2D, it allows us to have a clear perspective that the contact time reduction on rotating surfaces is dominated by the solid–liquid interplay during the droplet retracting stage, where *τ*_r_ decreases with both *v*_i_ and *ω*. For *v*_i_ = 1.60 m/s and *ω* = 9,000 rpm, *τ*_r_ can be reduced by up to ~50% compared with that on the stationary counterpart, which is slightly larger than that of the translational motion case (~40%) [37].

### Theoretical analysis

To elucidate the physical origin of the observed doughnut shape bouncing accompanied by the prominent contact time reduction, we make an analysis of the forces involved to describe the droplet dynamics. Because of the rotation of the underlying substrate, the impacting droplet is subject to an outward centrifugal force expressed as

$$F_{r}=\sum m_{\text{unit}}\omega^{2}R_{\text{unit}}={\rho \omega }^{2}\int_{0}^{H\left(t\right)}\int_{D_{\text{in}}\left(t\right)/2}^{D_{\text{out}}\left(t\right)/2}2\pi r^{2}\text{drdh}$$ (1)

where *m*_unit_ is the mass for an infinitely small volume element, *R*_unit_ is the corresponding radius of the volume element, *H*(*t*) is the integral upper limit for height *h*, and *D*_in_(*t*)/2 and *D*_out_(*t*)/2 are the integral lower and upper limit for radius *r*, respectively (Fig. S6). It is noticed that *D*_in_(*t*) always equals to zero before the formation of holes near the lamella center. Because of *F*_r_, the liquid tends to flow outward, so that the outer rim carries the dominant mass and momentum. Thus, the inner water lamella becomes very thin and easy to be destabilized because of the hydrodynamic instability, where *F*_r_ serves as a tensile stress to stretch the inner liquid molecules. Under the force *F*_r_ coupled with random disturbance (e.g., capillary waves), the spreading droplet on the high-speed rotating superhydrophobic surface is punctured at its thinnest region, through which the inner rim recoils simultaneously with the outer rim under the capillary action, and, finally, the droplet bounces off in a doughnut configuration. The doughnut-shaped bouncing is a unique outcome of a correlation between the droplet receding dynamics and the kinematics of surface rotation, which is counterintuitive on a macroscopically smooth surface despite the surface rotation. The physical mechanism discussed above also clearly elaborates the difference between droplet impact on rotating and translational moving surfaces [33,37–39]. During recoil, the droplet is also subject to a capillarity-induced retraction force $F_{c}=\ell \gamma \left(1-\min\theta_{R}\right)$ , where *θ*_R_ is the receding contact angle and ℓ is the length of triple-phase contact line. Before the nucleation of holes, ℓ can be estimated as ℓ ≈ *πD*_out_(*t*). After holes are generated, ℓ ≈ π[*D*_out_(*t*)+*D*_in_(*t*)], and, consequently, *F*_c_ increases dramatically compared to that on stationary surfaces. Furthermore, the average film thickness *h*_a_ can be obtained by combining the mass conservation scaling of $D_{\max}^{2}h_{a}\sim D_{0}^{3}$ and the experimental scaling of *D*_max_/*D*_0_ ~ We^1/3^ from Fig. 2C, which yields

$$h_{a}\sim {\left(D_{0}\gamma^{2}\rho^{-2}v_{i}^{-4}\right)}^{1/3}$$ (2)

Therefore, *h*_a_ scales inversely with *v*_i_. To initiate the hole nucleation, the thinnest region of the spreading lamella should be smaller than a critical film thickness h^*^, below which the liquid film is prone to destabilize and rupture. In this case, Eq. 2 reveals that a higher *v*_i_ endows a thinner *h*_a_, which makes the lamella vulnerable to break up under the rotation action. It is noted that the solid-liquid friction force is negligible. With a low contact angle hysteresis, the solid–liquid friction force is on the scale of about micronewtons, which is 2 to 3 orders of magnitude smaller than the dominated capillary force and centrifugal force.

From Fig. 2D, we find that the reduction of *τ* on rotating substrates is mainly determined by the interaction during the droplet retraction process. Thus, our attention is focused on *τ*_r_, which is essentially composed of 2 regimes (*τ*_r1_ and *τ*_r2_). In regime *τ*_r1_, only the outer rim participates in the recoil process. While in regime *τ*_r2_, the droplet recoils inward and outward simultaneously upon the appearance of holes, and *τ*_r2_ can be calculated as $\tau_{r2}\approx \ell_{r2}/\left(v_{\mathrm{ro}}+v_{\mathrm{ri}}\right)$, where ℓ_r2_ is the retraction length after the generation of holes. The retraction velocities of the inner rim (*v*_ri_) and outer rim (*v*_ro_) follow the Taylor–Culick retraction velocity [42], $v_{\mathrm{ri}}=\sqrt{2\gamma /\rho h_{i}}$ and $v_{\mathrm{ro}}\approx \sqrt{2\gamma /\rho h_{o}}$, where *h*_i_ and *h*_o_ are the specific thickness of inner and outer rims, respectively. Accordingly, *τ*_r2_ can be further expressed as $\tau_{r2}\approx \left(\ell_{r2}\sqrt{\rho }\right)/\left[\sqrt{2\gamma }\left(\sqrt{1/h_{o}}+\sqrt{1/h_{i}}\right)\right]$. For comparison, on stationary surfaces, $\tau_{r2}+\Delta \tau \approx \ell_{r2}\sqrt{\rho h_{o}}/\sqrt{2\gamma }$. Consequently, the contact time reduction Δ*τ* dictated by the rotating surfaces is given by $\Delta \tau \approx \ell_{r2}h_{o}\sqrt{\rho }/\left[2\gamma \left(\sqrt{h_{i}}+\sqrt{h_{o}}\right)\right]$. Assuming a linear relation of water film thickness as *h*_i_ = *mh*_o_ (0 < *m* < 1), where *m* represents a self-defined prefactor, the above expression of ∆*τ* can be simplified as

$$\Delta \tau \approx \ell_{r2}{\left(\frac{\rho h_{o}}{2\gamma }\right)}^{1/2}{\left(1+\sqrt{m}\right)}^{-1}$$ (3)

It suggests that the morphology of spreading droplets with a high thickness ratio between the inner and outer rims (i.e., larger *h*_o_ and smaller *m*) is expected to yield a larger contact time reduction. The thickness ratio is directly mediated by the defined physical parameter *ω*, considering that the rotating motion triggers radially outward symmetric fluid transport inside the spreading lamella that makes the inner rim thinner and the outer rim thicker.

To examine our model, we use the data corresponding to the rotating configuration in Fig. 2A, with measured ℓ_r2_ ~ 3.11 mm and *h*_o_~0.46 mm, to plot the theoretical contact time reduction ∆ *τ* against the prefactor *m* (Fig. S7). The experimental ∆*τ* ~ 4.3 ms allows an estimation of *m* ≈ 0.08, which is well within the above defined range (0 < *m* < 1). This estimation suggests that the inner rim is at least one order of magnitude thinner than the outer rim with the assistance of rotating motion. Together, the collective effects of *v*_i_ and *ω* result in the emergence of a new kind of doughnut-shaped bouncing with remarkable contact time reduction.

### Simulations

**Fig. 3.**
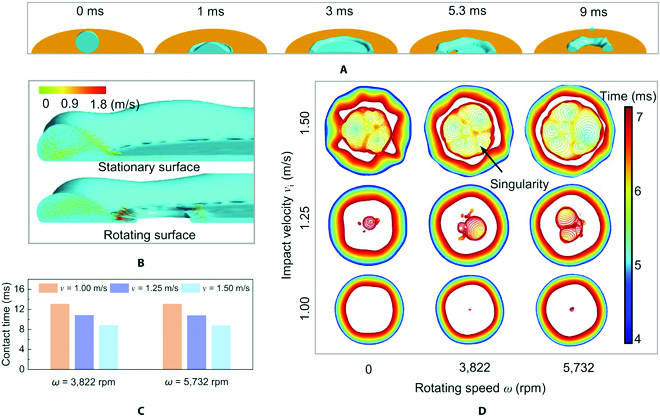
Numerical simulations. (A) The cross-sectional view snapshots of simulated droplet impact dynamics on rotating surfaces showing the presence of hole nucleatoin inside the spreading lamella (5.3 ms) and the final collision of inner and outer rims contact lines (9 ms). (B) Simulated results of velocity profiles in the cross section of the retracting rims for both stationary and rotating surfaces at 5.3 ms, clearly demonstrating that the inner rim on rotating surface recoils with a velocity of about 1.8 m/s, which is much faster than that of the outer rim (0.2 to 0.4 m/s). (C) Simulated contact time of impacting droplet on rotating surfaces as a function of *v*_i_ at the conditions of *ω* = 3,822 and 5,732 rpm. (D) Temporal evolution of contours of the triple-phase contact lines during the retraction stage under different *v*_i_ and *ω* values.

To quantify the fluid flow in the impacting droplet and the contact line dynamics, which are difficult to be visualized in the experiments, we further performed numerical simulations of droplet doughnut-shaped bouncing using the coupled level set and volume of fluid method (see Methods, Table S1, and Fig. S8). Figure 3A presents a few cross-sectional view images of the time-resolved evolution of droplet impacting on a rotating surface at *ω* = 5,732 rpm, which clearly display both the inner and outer rim dynamics. The numerical results are consistent with our experiments, where the droplet spreads, recoils, gets punctured, recedes from inner and outer rims simultaneously, and finally detaches the surface in a doughnut configuration with an overall contact time of about 9 ms (see also Movie S3). By extracting contours from the cross sections, it also clearly shows the thinning behavior of lamella with time during the receding period, which initiates the nucleation of hole (Fig. S9). Meanwhile, the enlarged flow velocity profile during the rim retraction at time 5.3 ms in Fig. 3B shows that the outer rim retracts with a velocity of 0.2 to 0.4 m/s toward the center, whereas the inner rim exhibits a much higher retraction velocity (up to ~1.8 m/s), which agrees to our experimental characterization in Fig. 2A. It is worth noting that the rotating droplet also shows a pronounced velocity component in the circumferential direction compared to the stationary surface (Fig. S10), which is endowed by the angular motion of the substrate. More interestingly, the collision by the inner and outer rims triggers a unique clockwise rolling behavior inside the doughnut-shaped droplet that has not been observed on stationary counterparts (Fig. S11).

To illustrate the effects of *v*_i_ and *ω* on the droplet dynamics and contact time reduction, from the perspective of numerical simulations, we calculate the contact time variations of different configurations, as shown in Fig. 3C. The increasing of *v*_i_ leads to a remarkable reduction of contact time, in consistency with our experimental observations. Moreover, Fig. 3D illustrates the temporal–spatial evolutions of the triple-phase contact lines during the droplet retraction process, with different colors representing different moments (see also Movie S4). The increasing of *ω* makes the liquid film more vulnerable to be punctured, thereby the amount of singularities that initiate the hole nucleation rises (marked black arrow in Fig. 3D). It is noted that although the singularity-triggered holes appear with random distributions in the spreading lamella, they eventually coalesce into a larger one. Besides, by increasing *v*_i_, nucleated holes are prone to appear at an earlier time, and thus, the retraction process of inner rims can start earlier, leading to much larger contact time reduction.

Apart from the velocity vectors and contact line evolution, we also investigate the spatial and temporal distribution of liquid pressure on high-speed rotating superhydrophobic surfaces. (Fig. S12). At the beginning of the retraction stage (4 ms), an outside-inside pressure gradient is observed, driving the outer rim to recoil. Because of the tensile stress induced by the centrifugal force *F*_r_, several spots with negative pressure emerge (5 ms), at which the lamella breaks up (5.3 ms). Afterward, a rather high pressure gradient is generated in the vicinity of inner rim edge, which promotes the fast expansion of holes (5.3 and 5.7 ms). When the doughnut-shaped configuration appears (6.8 ms), the overall pressure distribution inside the droplet tends to be relatively uniform, suggesting the completion of in-plane retraction and onset of out-of-plane momentum for bouncing.

### Versatility of contact time reduction

**Fig. 4.**
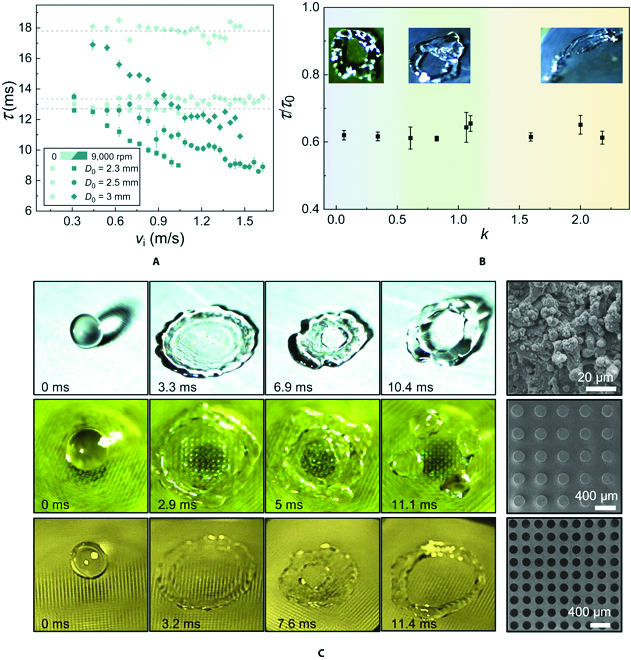
Versatility of contact time reduction on rotating surfaces. (A) The demonstration of contact time reduction on rotating surfaces compared with stationary surfaces for different droplet diameters of 2.3, 2.5, and 3 mm, respectively. (B) The normalized contact time *τ*/*τ*_0_ is independent of the landing locations on rotating surfaces, although different droplet bouncing dynamics are observed. (C) The doughnut-shaped bouncing on rotating surfaces can be generalized to a wide range of materials and structures. From top to bottom: Nanostructure aluminum, 3D printed resin micropillars, and 3D printed resin microholes. The rightmost column illustrates the scanning electron microscopy images of the employed substrates for each panel.

The doughnut-shaped bouncing-induced contact time reduction can be generalized to a broad range of working conditions. Here, we further conducted experiments with different droplet sizes, landing locations, and substrate materials and/or structures. As shown in Fig. 4A, the contact time reduction is also valid for droplets of diameters *D*_0_ ranging from 2.3 to 3 mm. More importantly, the results reveal that the decreasing trend of *τ* as a function of *v*_i_ could be generalized to various droplet sizes. Figure 4A also indicates that under the rotation effect, *τ* increases with the increasing of *D*_0_, which is consistent with the situation for stationary surfaces, considering that a larger droplet size usually means a longer recoiling distance. To examine the landing location effect, we define a dimensionless off-centered factor *k*, representing the horizontal distance between the center of rotating surfaces and the centroid of the impacting droplet ∆*L*, normalized by its diameter *D*_0_, or *k* = ∆*L*/*D*_0_. The variation of dimensionless contact time τ^*^ as a function of *k* is then plotted in Fig. 4B, which clearly demonstrates 3 distinct regimes. In the regime near the rotating center (0 ≤ *k* ≤ 0.5), the droplet can bounce in a doughnut configuration, analogous to that shown in Fig. 1C. However, in the regime far away from the center with *k* > 1.25, the droplet spreads asymmetrically and rebounds in an elongated manner. In this case, the reduced contact time is attributed to the asymmetric retraction, which is somewhat like previous works on translational moving surfaces [36,37]. Between these 2 regimes, we also observe a transitional regime where the doughnut-shaped and asymmetric bouncing are coexistent, so that the droplet displays an elongated fashion along with an off-centered hole.

In particular, our investigation bridges the gap between the unexplored rotation effect and the well-studied translational moving effect on the contact time reduction of impacting droplets. Despite dissimilar bouncing dynamics for different landing locations, the contact time reduction behavior of impacting droplets is universal, which is strongly mediated by the rotating surface. Besides, the universality also exists for the dependence of *τ* on *v*_i_, as exemplified for one case with *k* ≈ 2 shown in Fig. S13. The generality of the doughnut-shaped bouncing behavior is further examined by experiments on various substrate materials and structures, including nanostructured aluminum, three-dimensional (3D) printed resin with micropillars, and 3D printed resin with microholes (Fig. 4C and Methods). The impacting droplet dynamics on these surfaces are reminiscent of that on the CuO nanostructured surface (Fig. 1C).

## Conclusion

In summary, we have experimentally, numerically, and theoretically explored how the high-speed rotation motion of superhydrophobic surfaces reshapes the droplet dynamics. Remarkably, we discover a symmetric doughnut-shaped bouncing phenomenon of droplets accompanied by their spontaneous scattering. The rotation of the substrate substantially reduces the contact time between the droplet and the solid, which results from the accelerated retraction process by the centrifugal force induced additional receding contact line. We found that the conventional inertial-capillary scaling law of $\tau \sim {\left(\rho D_{0}^{3}/\gamma \right)}^{1/2}$ is not applicable to our case for regime of *v*_i_ > *v*_t_, as a new physical parameter *ω* is introduced; instead, *τ* decreases with the increasing of *v*_i_. The spectacular droplet dynamics can be generalized to a wide scope of working conditions for different droplet sizes, land locations, and substrate materials/structures. This work not only advances the fundamental understanding of classical wetting dynamics but also suggests an efficient methodology to actively control the contact time of droplets impacting on solid surfaces.

## Methods

### Experimental setup

Experiments were performed in ambient environment at room temperature with ~50% relative humidity. The apparatus was placed on a vibration isolation platform. Two light-emitting diode lamps are applied for illumination. Deionized water droplets with diameters *D*_0_ ranging from 2.3 to 3 mm were created by stainless steel needles with different sizes, connected to a 20-ml plastic syringe and a syringe pump (LSP01-3A) via a rubber tube, from a predetermined height. The sample was placed on the center of a spin coater and absorbed to the base by vacuum. The angular velocity of the spin coater, changed from 1,000 to 9,000 rpm, can be directly adjusted at the control panel. Two high-speed cameras (Photron FASTCAM Nova S12 and Mini AX200) were synchronized to capture the droplet impact dynamics from both side and top views at a frame rate of 10,000 frames per second with a shutter speed of 1/10,000 s. The images were analyzed by the software Photron Fastcam Viewer, MATLAB, and ImageJ.

### Sample fabrications

#### Nanostructured copper oxide

Copper samples were first ultrasonically cleaned by acetone, IPA, and ethanol, and then they were washed by the deionized water thoroughly. This was followed by a dip in the diluted hydrochloric acid (1 M) for 10 s to remove the native oxide. The nanostructured CuO surface was created by immersing the clean copper samples in a freshly mixed aqueous solution of NaClO_2_, NaOH, Na_3_PO_4_·12H_2_O and deionized water (3.75:5:10:100) at 95 °C for 1 h. During the oxidation process, the sharp-knife-like copper oxide nanostructures were formed. Subsequently, a thorough rinsing with deionized water followed by a complete drying in a nitrogen stream was conducted. Finally, the surface wettability was modified by silanization treatment in a 1 mM *n*-hexane solution of trichloro(1*H*,1*H*,2*H*,2*H*-perfluorooctyl) silane for 60 min and then heated at 150 °C in air for 30 min to render surfaces superhydrophobic.

#### 3D printed micropillars and microholes

The resin sample was built by a projection-microstereolithography-based 3D printing technique (BMF Precision Tech Inc., nanoArch S140) with 10-μm resolution. A model was prebuilt by a mapping software and then sliced into a suite of 2D images of predetermined thickness, followed by an exposure process. The diameter and center-to-center spacing of micropillars were 200 and 450 μm, respectively. For microholes, the diameter and center-to-center spacing were 170 and 250 μm, respectively. The above steps were iterated for each layer until the designed structure was created. A similar silanization treatment using trichloro(1*H*,1*H*,2*H*,2*H*-perfluorooctyl) silane was performed. Finally, the surface was spray-coated by a commercial nanoparticle dispensed solution (Glaco, Soft 99 Co.) and heated at 100 °C for 60 min.

#### Nanostructured aluminum

The aluminum samples were first ultrasonically cleaned by acetone, isopropyl alcohol, ethanol, and deionized water. To create random nanostructures, the cleaned aluminum samples received laser treatment (YLP-50-IPG) at 1,030-nm wavelength and 25-W maximum continuous output. The laser beam was scanned along the surface sample with a constant speed of 1,200 mm/s. The repetition rate is 50 kHz, and the sample was maintained normal to the direction of the incident beam. To ensure a sufficient material removal, the constructing process was repeated 5 times. Finally, the as-prepared samples were immersed in a 1 mM hexane solution of trichloro(1*H*,1*H*,2*H*,2*H*-perfluorooctyl) silane for 60 min, followed by a heat treatment for drying.

### Characterizations

To characterize the surface wettability, the static contact angle on the nanostructured CuO surfaces was measured by a contact angle goniometer (Dataphysics Instrument OCA 25). A deionized water droplet of 8.5 μl was deposited on the substrate surface at room temperature with ~50% relative humidity. The static, advancing, and receding contact angles were measured to be 161° ± 1°, 164° ± 3°, and 157° ± 1°, respectively, illustrating excellent nonwetting property. Five independent measurements were carried out to obtain the average contact angles. The surface morphology was investigated by a field-emission scanning electron microscopy (ZEISS SUPRA 55) at 10 kV.

### Numerical methods

The numerical calculations were conducted in software ANSYS Fluent. The dynamics of water droplets impacting on stationary and rotating surfaces were both simulated using the coupled level set and volume of fluid method.

Water and air were simulated within a cylindrical domain with a radius of 6.0 mm and a height of 12.0 mm, which contains more than 2.4 million prismatic cells generated by a sweeping method after grid independence tests. To improve the accuracy of calculations, the near-wall grid was densified self-adaptively. The corresponding time step, 1.0 μs, is of high efficiency and convergence. The bottom surface was treated as a moving wall with different rotating speeds, and the other surfaces of the cylindrical domain were set as pressure outlet boundaries. The coupling equations of pressure and velocities were solved via the pressure implicit split operator method. As for the 2 phases, the primary one was set as air, while the secondary one was set as water. At the initial stage, the water droplet was modeled as a sphere with a downward impact velocity. All parameters used in the simulations are listed in Table S1.

## Data Availability

All data supporting the findings of this study are available within the paper and its supplementary information.

## Supplementary Materials

Supplementary 1 Figs. S1 to S13 and Table S1 show simulation parameters, experimental setup, and additional data for experiments and simulations, including droplet kinematics, dynamics, contours, and distributions of pressure and velocity vectors (PDF). Movies S1 to S4 show doughnut-shaped droplet bouncing dynamics. Table S1. List of simulation parameters. Fig. S1. Experimental setup. Fig. S2. Droplet impact dynamics on rotating hydrophilic surfaces. Fig. S3. Temporal evolution of spreading length of droplet on a rotational superhydrophobic surface with ω = 9,000 rpm in a wide time range, which is divided into 3 regimes. Fig. S4. Phase diagram revealing the occurrence of contact time reduction by changing We (or *v*_i_) and *ω*. Fig. S5. Contact time τ as a function of impact velocity *v*_i_ under various angular velocity *ω*. Fig. S6. Schematic illustration of droplet contours before and after the rupture, respectively. Fig. S7. The variation of contact time reduction Δ*τ* as a function of m based on the proposed theoretical model described as Eq. 3 in the main text, where the shaded areas represent one standard deviation of the data. Fig. S8. 3D computational domain and structured meshes. Fig. S9. Contours of droplet cross section during retractions by numerical calculation. Fig. S10. Top-view velocity vectors of a rotating droplet at *ω* = 5,732 rpm. Fig. S11. Velocity vectors inside the doughnut-shaped droplet, demonstrating a clockwise rolling behavior, which is caused by the unbalanced momentums between the inner and outer rims at collision. Fig. S12. Temporal variation of pressure distributions of the doughnut-shaped droplet on the high-speed rotating surface (*ω* = 5,732 rpm) before bouncing. Fig. S13. Contact time *τ* as a function of impact velocity *v*_i_ under various angular velocity *ω* at off-centered condition (*k* ≈ 2).

Supplementary 2 Movie S1. Side view of droplet doughnut-shaped bouncing on high-speed rotating surfaces with *v*_i_ = 1.56 m/s and *ω* = 9,000 rpm.

Supplementary 2 Movie S2. Top view of droplet doughnut-shaped bouncing on high-speed rotating surfaces with *v*_i_ = 1.56 m/s and *ω* = 9,000 rpm.

Supplementary 2 Movie S3. Numerical simulation of droplet doughnut-shaped bouncing on high-speed rotating surfaces with *v*_i_ = 1.5 m/s and *ω* = 5,732 rpm.

Supplementary 2 Movie S4. Numerical simulation of contact line temporal evolution on high-speed rotating surfaces with *v*_i_ = 1.25 m/s and *ω* = 3,822 rpm.
