# Supplementary material for: Rotating Surfaces Promote the Shedding of Droplets
Source: Research (Wash D C). 2023 Jan 10;6:0023. doi: 10.34133/research.0023 (PMC10076004; doi:10.34133/research.0023)
Supplement: Supplementary 1 — Figs. S1 to S13 and Table S1 show simulation parameters, experimental setup, and additional data for experiments and simulations, including droplet kinematics, dynamics, contours, and distributions of pressure and velocity vectors (PDF). Movies S1 to S4 show doughnut-shaped droplet bouncing dynamics. Table S1. List of simulation parameters. Fig. S1. Experimental setup. Fig. S2. Droplet impact dynamics on rotating hydrophilic surfaces. Fig. S3. Temporal evolution of spreading length of droplet on a rotational superhydrophobic surface with ω = 9,000 rpm in a wide time range, which is divided into 3 regimes. Fig. S4. Phase diagram revealing the occurrence of contact time reduction by changing We (or vi) and ω. Fig. S5. Contact time τ as a function of impact velocity vi under various angular velocity ω. Fig. S6. Schematic illustration of droplet contours before and after the rupture, respectively. Fig. S7. The variation of contact time reduction Δτ as a function of m based on the proposed theoretical model described as Eq. 3 in the main text, where the shaded areas represent one standard deviation of the data. Fig. S8. 3D computational domain and structured meshes. Fig. S9. Contours of droplet cross section during retractions by numerical calculation. Fig. S10. Top-view velocity vectors of a rotating droplet at ω = 5,732 rpm. Fig. S11. Velocity vectors inside the doughnut-shaped droplet, demonstrating a clockwise rolling behavior, which is caused by the unbalanced momentums between the inner and outer rims at collision. Fig. S12. Temporal variation of pressure distributions of the doughnut-shaped droplet on the high-speed rotating surface (ω = 5,732 rpm) before bouncing. Fig. S13. Contact time τ as a function of impact velocity vi under various angular velocity ω at off-centered condition (k ≈ 2). [file research.0023.f1.pdf]

Rotating surfaces promote the shedding of droplets

Ran Tao,[1,‡] Wei Fang,[2,‡] Jun Wu,[1] Binhong Dou,[1] Wanghuai Xu,[3] Zhanying Zheng,[1] Bing Li,[1] Zuankai Wang,[3,] Xiqiao Feng,[2,*] Chonglei Hao[1,*]*

[1] School of Mechanical Engineering and Automation, Harbin Institute of Technology, Shenzhen 518055, China

[2] Institute of Biomechanics and Medical Engineering, Applied Mechanics Laboratory, Department of Engineering Mechanics, Tsinghua University, Beijing 100084, China

[3] Department of Mechanical Engineering, City University of Hong Kong, Hong Kong 999077, China

[‡] These authors contributed equally to this work.

[*] To whom correspondence should be addressed: haoc@hit.edu.cn; fengxq@tsinghua.edu.cn; zuanwang@polyu.edu.hk

Table S1. List of simulation parameters.

**Parameters**	**Values**	**Units**
Water drop diameter	2.5	mm
Drop impact velocity	1.0 ~ 1.5	m s^{-1}
Density of the water, air	998.0, 1.225	kg m^{-3}
Viscosity of the water, air	1.003e−3, 1.789e−5	Pa s
Gravitational acceleration	9.8	m s^{-2}
Surface tension of the water	72	mN m^{-1}
Contact angle of the SHBS	160	°
Rotational speed of the SHBS	0 ~ 600	rad s^{-1}

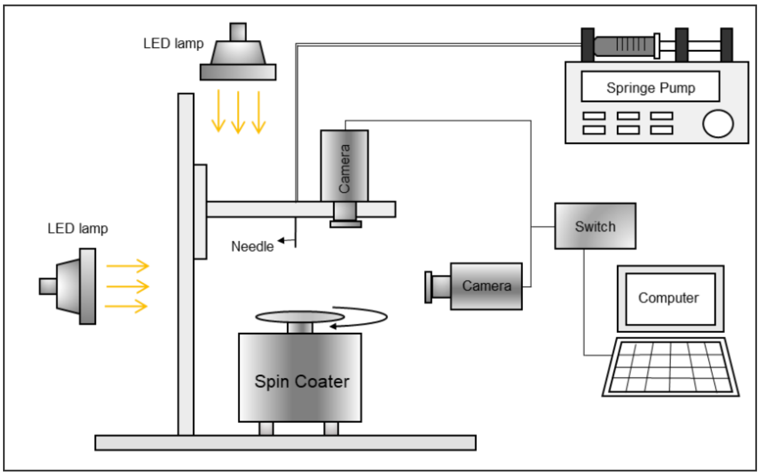

Figure S1. Experimental setup. Deionized water droplet was created by stainless steel needles with different sizes, connected to a syringe and a syringe pump via a rubber tube, from a pre-determined height. The sample was placed on the center of a spin coater and absorbed to the base by vacuum. The angular velocity of the spin coater can be directly adjusted at the control panel. Two high-speed cameras were synchronized to capture the droplet impact dynamics from both side and top views.

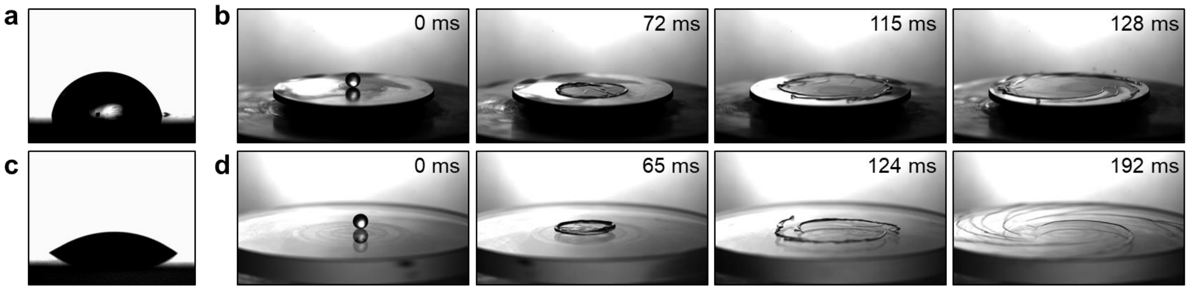

Figure S2. Water droplet impact dynamics on rotating hydrophilic surfaces (a) Droplet deposited on smooth copper substrate exhibiting a static contact angle of ~ 86°. (b) Selected snapshots showing the droplet impact dynamics with a velocity v_i = 1.56 m/s on a rotating copper surface (ω = 9,000 rpm). (c) Droplet deposited on smooth glass substrate exhibiting a static contact angle of ~ 30°. (d) Selected snapshots showing the droplet impact dynamics with a velocity v_i = 1.56 m/s on a rotating glass surface (ω = 9,000 rpm).

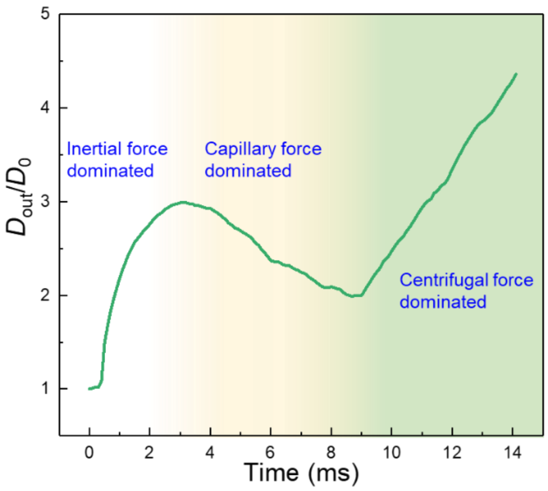

Figure S3. Temporal evolution of spreading length of droplet on a rotational SHPS with ω = 9000 rpm in a wide time range, which is divided into three regimes. In the first regime (white area), the droplet spreads to maximum, dominated by the inertia force. In the second regime (yellow area), the droplet retracts owing to the capillary effect and bounces off at the end of this regime. In the third regime (green area), the increase of the outer rim diameter D_{out} indicates that the lifting-off droplet keep expanding, suggesting a centrifugal force dominated kinematics and a spontaneous liquid removal.

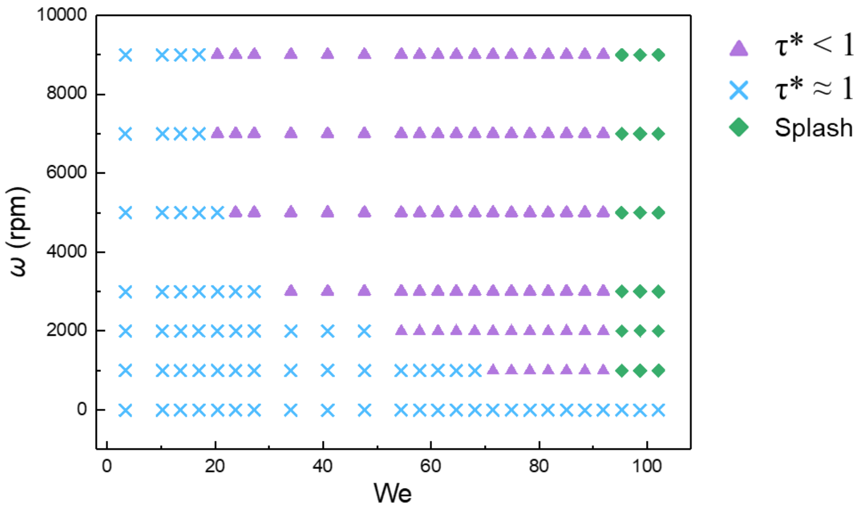

Figure S4. Phase diagram revealing the occurrence of contact time reduction by changing We (or v_i) and ω. When both We and ω are small, no contact time reduction is observed. At intermediate We and ω, the droplet bounces with a reduced sold-liquid contact time. The lowering of We or ω can be compensated by an excess of another. Beyond a critical We, the droplet breaks up on rotating surfaces. The phase diagram indicates that the behavior of contact time reduction could be optimized by tailoring kinetic parameters.

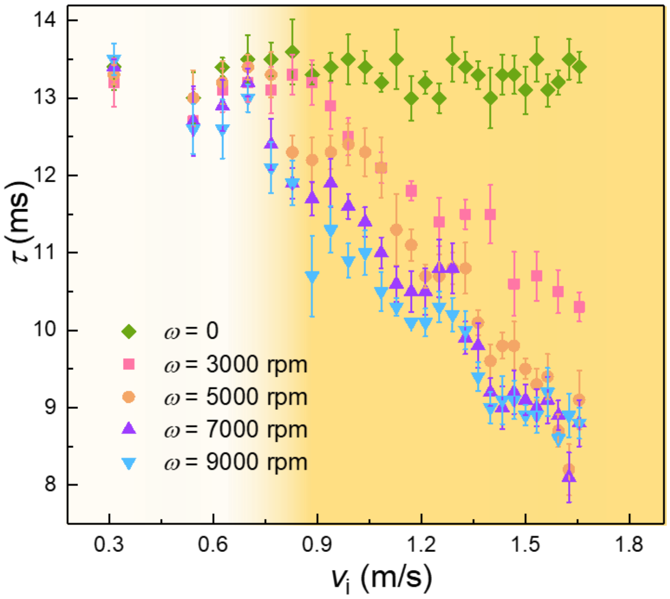

Figure S5. Contact time τ as a function of impact velocity v_i under various angular velocity ω. Generally, the data falls into two regimes and the transition is characterized by a threshold velocity v_t, which ranges between 0.70 m/s to 0.94 m/s, depending on the angular velocity ω. At the regime of $v_i < v_t$, the contact time on rotating surfaces is roughly close to that on the stationary counterpart. Beyond v_t, τ is decreased from ~13.5 ms to nearly 8 ms.

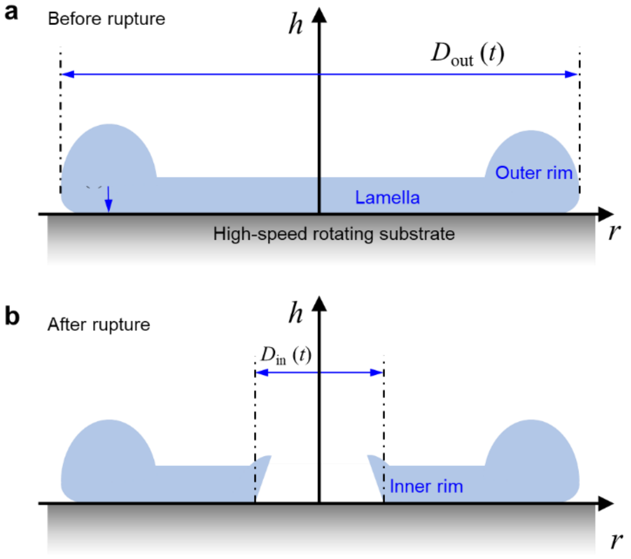

Figure S6. Schematic illustration of droplet contours (a) before and (b) after the rupture. Before rupture, the droplet is composed of the lamella and the outer rim. The diameter and the height of the outer rim is denoted by D_{out} (t) and $H(t)$, respectively. After the rupture of the lamella, a hole is nucleated accompanied by a newly generated inner rim, which retracts simultaneously with the outer rim. The diameter of the hole is represented by D_{in} (t).

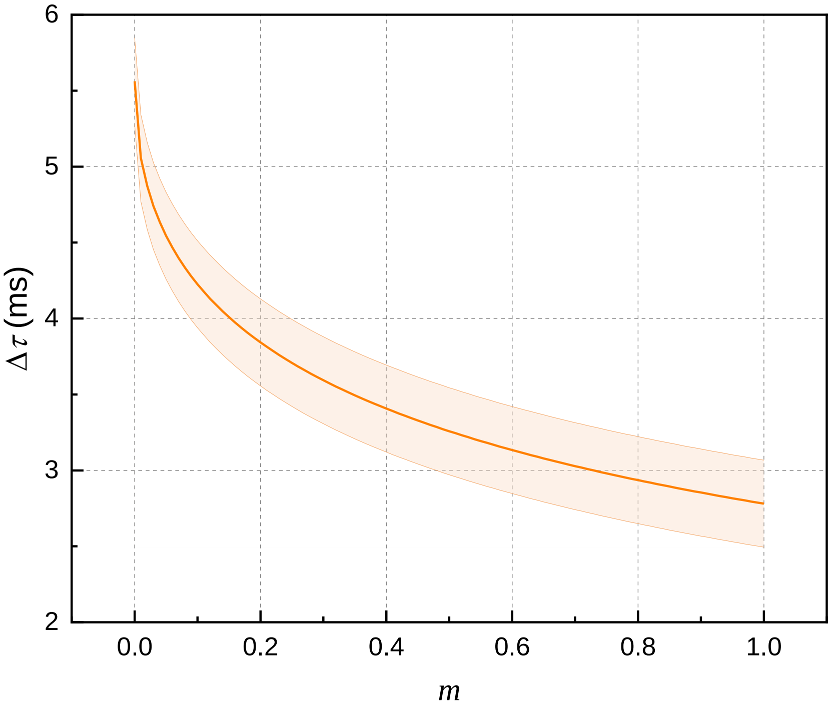

Figure S7. The variation of contact time reduction $\Delta\tau$ as a function of m based on the proposed theoretical model described as Eq. (3) in the main text, where the shaded areas represent one standard deviation of the data. For a typical $\Delta\tau$ of ~ 4.3 ms, the corresponding value of m is estimated to be about 0.08, indicating that the inner rim is at least one order of magnitude thinner than the outer rim.

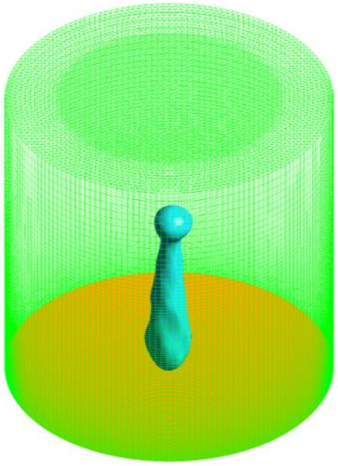

Figure S8. Three-dimensional computational domain and structured meshes. The red region represents superhydrophobic surfaces (SHPS) and blue region represent droplets.

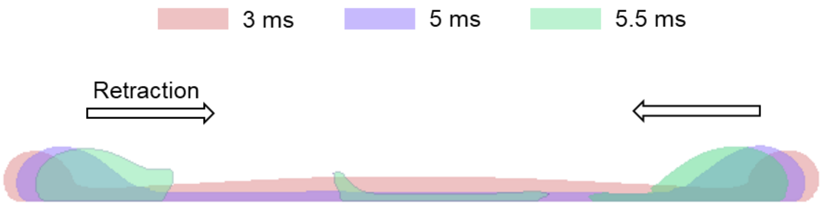

Figure S9. Contours of droplet cross-section during retractions by numerical calculation. At the initial stage of retraction, the lamella becomes thinner while the outer rims become thicker and finally the lamella ruptures at its thinnest region. The time sequences of contours highlighted by green, violet, and pink colors are 3 ms, 5 ms and 5.5 ms.

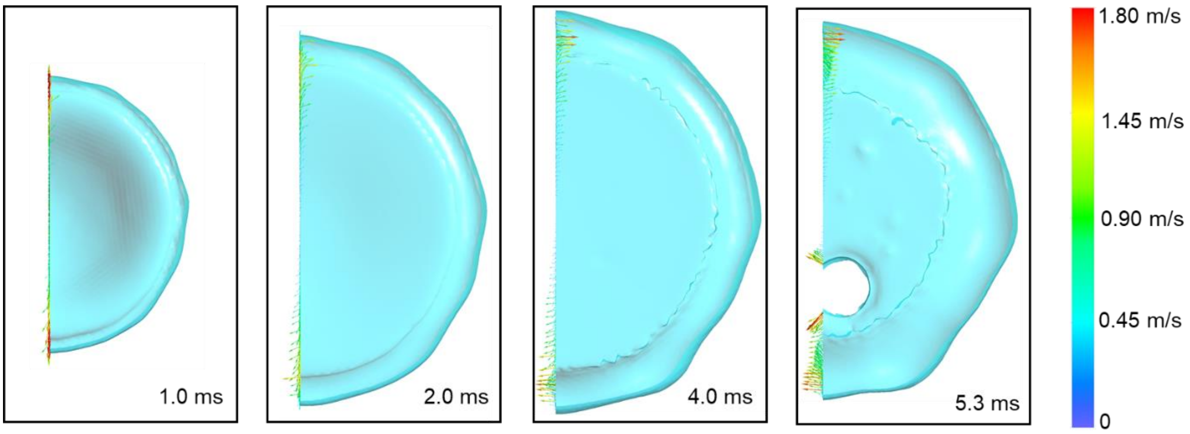

Figure S10. Top view velocity vectors of a rotating droplet at ω = 5732 rpm. At the initial stage of spreading, the tangential velocity is weak and the radial velocity is dominant due to the limited interaction between the liquid and the rotating substrate. Over time, a pronounced circumferential velocity is observed although it is slightly manipulated by the rim velocity (either spreading or retraction). Once the hole forms, the velocity vector of the inner rim is deflected by coupling the circumferential velocity and the fast retraction velocity.

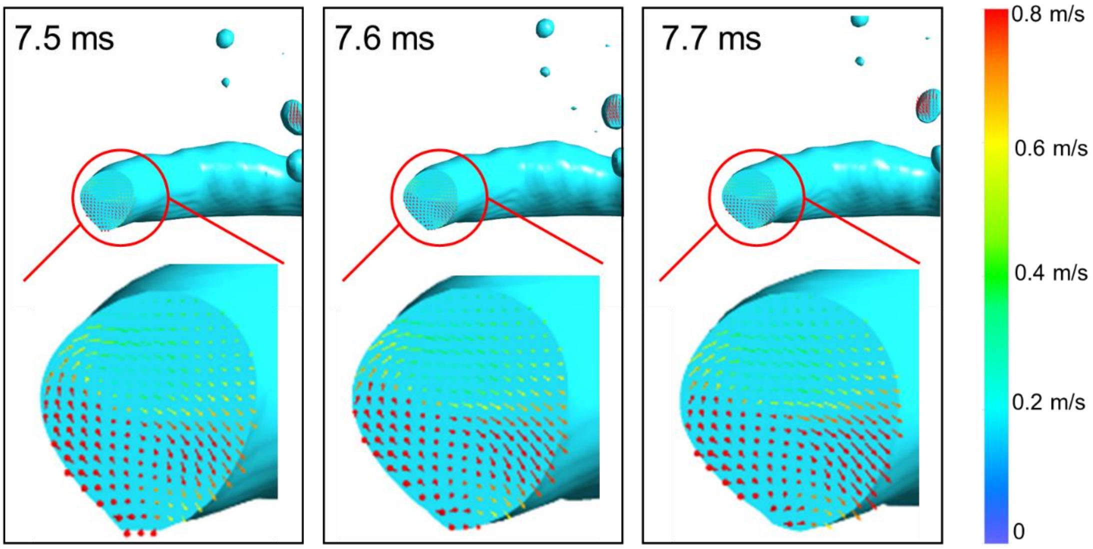

Figure S11. Velocity vectors inside the doughnut-shaped droplet, demonstrating a clockwise rolling behavior, which is caused by the unbalanced momentums between the inner and outer rims at collision. The unique rolling manner has not been observed on stationary counterparts.

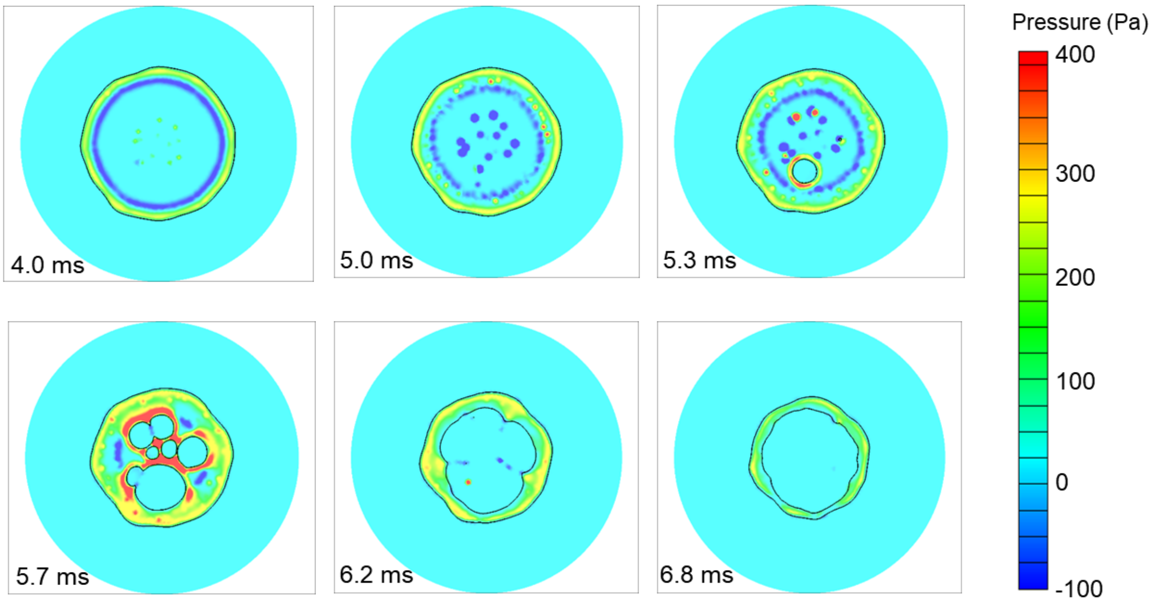

Figure S12. Temporal variation of pressure distributions of the doughnut-shaped droplet on the high-speed rotating surface (ω = 5,732 rpm) before bouncing. At 4 ms, an evident pressure gradient along the droplet outer rim is observed so the droplet retracts inward. At 5 ms, several spots with negative pressures appear under the tensile force executed by F_r. The holes are easy to form at the negative pressure spots. The nucleation of holes is accompanied by a high pressure gradient along the edge of holes (5.3ms and 5.7ms). The pressure distribution is relatively uniform when the droplet exhibits the doughnut shape (6.8 ms), which suggests the completion of in-plane retraction and onset of out-of-plane momentum for bouncing.

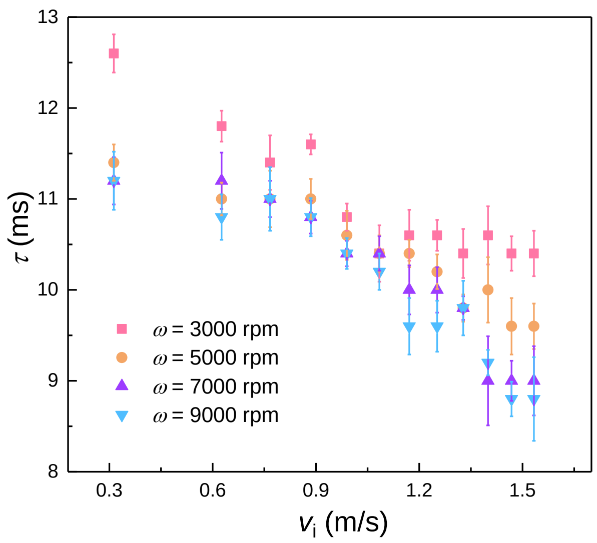

Figure S13. Contact time τ as a function of impact velocity v_i under various angular velocity ω at off-centered condition ($k \approx 2$). At low v_i, the variation of τ is small while at high v_i, τ is monotonically decreased with the increasing of v_i. The trend is similar to that droplet landing on the center of the rotating substrate.

Supplementary Movies

Supplementary Video S1

Description: Side view of droplet doughnut-shaped bouncing on high-speed rotating surfaces with v_i = 1.56 m/s and ω = 9,000 rpm.

Supplementary Video S2

Description: Top view of droplet doughnut-shaped bouncing on high-speed rotating surfaces with v_i = 1.56 m/s and ω = 9,000 rpm.

Supplementary Video S3

Description: Numerical simulation of droplet doughnut-shaped bouncing on high-speed rotating surfaces with v_i = 1.5 m/s and ω = 5,732 rpm.

Supplementary Video S4

Description: Numerical simulation of contact line temporal evolution on high-speed rotating surfaces with v_i = 1.25 m/s and ω = 3,822 rpm.
